# Measurement Invariance of the Kessler Psychological Distress Scale (K-10) Among Chilean, Colombian and Ecuadorian University Students

**DOI:** 10.3390/bs16101812

**Published:** 2026-10-03

**Authors:** Ignacio Norambuena-Paredes, José Luis Gálvez-Nieto, Eulalia Elizabeth Salas-Tenesaca, Cristina Tavera-Cuellar, Constanza Norambuena-Candia

**Affiliations:** 1 Departamento de Trabajo Social, Universidad de La Frontera, Temuco 4780000, Chile; ignacio.norambuena@ufrontera.cl; 2 Departamento de Ciencias Empresariales, Universidad Técnica Particular de Loja, Loja 110107, Ecuador; eesalas@utpl.edu.ec; 3 Facultad de Administración de Empresas, Universidad Santo Tomás Bucaramanga, Bucaramanga 681011, Colombia; cristina.tavera@ustabuca.edu.co; 4 Programa Magister en Educación Física y Vida Saludable, Universidad de La Frontera, Temuco 4780000, Chile; c.norambuena01@ufromail.cl

**Keywords:** psychological distress, mental health, measurement invariance, university students, Latin America

## Abstract

Psychological distress among university students requires measures that function equivalently across cultural contexts. This instrumental study evaluated the factorial structure, measurement invariance, convergent validity, and reliability of the Kessler Psychological Distress Scale (K-10) in 1258 students from Chile (456), Colombia (319), and Ecuador (483). Confirmatory factor analyses were estimated with WLSMV for ordered categorical indicators. Because the original five-category model showed inadequate fit, alternative recodings were examined. A binary solution distinguishing never or almost never from symptoms experienced at least occasionally was retained and showed adequate unidimensional fit in each country. Multigroup analyses supported configural and scalar invariance across the three samples. Latent mean comparisons showed higher psychological distress in Ecuador than in Chile and Colombia, whereas Chile and Colombia did not differ significantly. Psychological distress was inversely associated with life satisfaction in each country, providing evidence of convergent validity. Ordinal omega coefficients ranged from 0.910 to 0.936, indicating high internal consistency. Overall, the findings support the use of the binary K-10 as a dimensional measure of psychological distress for cross-cultural research and group-level comparisons within the participating samples. However, generalisation to the original response format and use for individual screening, clinical decision-making, intervention evaluation, or monitoring change require additional validation.

## 1. Introduction

Contemporary society is characterised by profound social, economic, cultural, and technological changes that have transformed the ways in which individuals relate to themselves, others, and their surrounding environment (Mohamed et al., 2025; Norambuena-Paredes, 2025; Sheripova & Alieva, 2024). Globalisation, the digitalisation of everyday life, and the increasing pace of social change have created contexts marked by uncertainty, competitiveness, and the constant need for adaptation (Ball et al., 2024; Han, 2014; Sing-Yun, 2023). Within this landscape, demands associated with personal, academic, and professional performance have become increasingly prominent, generating new forms of pressure that directly affect individuals’ quality of life and well-being (Gálvez-Nieto et al., 2025; Davinson-Pacheco et al., 2026; Polanco-Levicán et al., 2025). As a result, mental health has become a major concern for researchers, institutions, and public organisations seeking to understand the challenges faced by contemporary societies (De Oliveira et al., 2025; Gu, 2024; Nykiforuk et al., 2024).

These transformations have had a particularly significant impact on young people, who are at a developmental stage characterised by identity formation and the construction of their personal and professional futures (Deutz, 2023; Fornell et al., 2023; Sevilla-Santo et al., 2021). In this context, higher education provides opportunities for academic and social development, while simultaneously constituting an environment in which multiple demands and challenges converge (Polanco-Levicán et al., 2023; Troncoso-Tejada et al., 2026; Yaping et al., 2023). The need to meet increasing academic demands, achieve performance goals, and balance multiple responsibilities may generate experiences of stress that adversely affect university students’ psychological well-being (Alwarthan et al., 2022; Norambuena-Paredes et al., 2025). Consequently, the university years represent a particularly sensitive period for the study of mental health (Aghabozorgi et al., 2024; Hutchesson et al., 2025; Paduano et al., 2025).

Within this framework, psychological distress has emerged as a key construct for understanding the emotional suffering experienced by individuals when confronted with situations perceived as demanding or difficult to manage (Ben Brik et al., 2024; Katsikidou et al., 2025). This construct refers to a general state of emotional impairment characterised by symptoms related to anxiety, excessive worry, sadness, exhaustion, and a perceived inability to cope effectively with everyday demands (Fairbrother et al., 2025; Łagowska & Ptaszyńska, 2026). Unlike clinically diagnosed mental disorders, psychological distress represents a broader and more non-specific condition that may vary in intensity and affect multiple dimensions of individual functioning (Dwight et al., 2024; Lin et al., 2023).

From a comprehensive perspective, psychological distress arises from the interaction of personal, social, and contextual factors (Cooper et al., 2025; Owens et al., 2024). Stressful experiences, financial difficulties, interpersonal conflicts, social expectations, and pressures stemming from different areas of life may contribute to its onset and persistence (Maldonado et al., 2022; Uzun & Arslan, 2025). Likewise, the availability of coping resources, social support, and favourable conditions for personal development plays a fundamental role in how individuals respond to adverse circumstances (Kjellstrand et al., 2023; Sujiv et al., 2022). Therefore, psychological distress should be understood as a multidimensional phenomenon that extends beyond individual characteristics and is closely linked to the social conditions in which people live (Ferreira et al., 2021; Vaswani et al., 2022).

Among university students, psychological distress is of particular importance because of the unique characteristics of the academic experience (Wood et al., 2024; Zeng et al., 2024). Entering higher education involves confronting new challenges related to autonomy, adapting to unfamiliar environments, establishing social networks, managing time effectively, and fulfilling academic responsibilities (Gálvez-Nieto et al., 2025; Polanco-Levicán et al., 2026; Troncoso-Tejada et al., 2026). In addition, concerns regarding future employment, family expectations, and academic performance demands may increase perceptions of stress and vulnerability (García-García & Carrizales-Berlanga, 2021; Norambuena et al., 2025; Satizabal-Reyes et al., 2023). These circumstances make university students a population that is particularly vulnerable to experiencing different manifestations of psychological distress (Baumhauer et al., 2025).

Psychological distress among university students has been associated with numerous adverse consequences at both the individual and academic levels (Cassaretto et al., 2021; Flynn et al., 2024; Zhai et al., 2026). These include reduced life satisfaction, impaired concentration, poorer academic performance, deterioration in interpersonal relationships, and diminished capacity to cope with complex situations (Arslan et al., 2025; Peixoto & Cunha, 2024; Rahimi et al., 2025). Similarly, elevated levels of psychological distress may undermine motivation, academic engagement, and persistence in higher education (Jones et al., 2022; Mahdavi et al., 2023). Consequently, understanding the factors associated with this phenomenon is essential for promoting successful academic trajectories and enhancing students’ overall well-being (Liang et al., 2026; Mayberry et al., 2022).

Given the growing concern surrounding mental health within higher education settings, the study of psychological distress has become a priority research area across the social sciences, psychology, and higher education. Investigating this phenomenon provides insight into how students experience and respond to the demands of university life, enabling the identification of both risk and protective factors that influence their well-being. Furthermore, generating empirical evidence on psychological distress supports the development of institutional strategies aimed at strengthening mental health, promoting healthy educational environments, and fostering the holistic development of future professionals.

### Kessler Psychological Distress Scale (K-10): Description and Theoretical Structure of the Construct

The Kessler Psychological Distress Scale (K-10) was developed by Kessler et al. (2002) to provide a brief, sensitive, and psychometrically robust instrument for monitoring the prevalence and trends of psychological distress in population-based studies. Its development was based on the identification of non-specific symptoms associated with anxiety and depression, which were conceptualised as indicators of a general factor of psychological distress. From this perspective, the K-10 assesses emotional, cognitive, and psychophysiological symptoms experienced during the previous 30 days, providing a dimensional measure of psychological distress rather than a specific clinical diagnosis (Kessler et al., 2002, 2003). The theoretical framework underpinning the instrument is based on the existence of a unidimensional latent construct reflecting general emotional vulnerability, which has been shown to be closely associated with a range of common mental disorders.

From a theoretical perspective, the construct of psychological distress assessed by the K-10 can be understood as a non-specific emotional response to stress, adversity, or difficulties in adaptation, characterised by the simultaneous presence of symptoms associated with anxiety and depression. This conceptualisation is grounded in the transactional model of stress proposed by Lazarus and Folkman (1984), which posits that psychological well-being depends on individuals’ cognitive appraisal of environmental demands and the resources available to cope with them. When such demands are perceived as exceeding available coping resources, the likelihood of experiencing emotional strain, hopelessness, exhaustion, and negative affect increases.

Complementing this perspective, Mirowsky and Ross (2002) argue that psychological distress represents a general indicator of emotional suffering resulting from exposure to adverse social, economic, and interpersonal conditions. Consistent with these theoretical perspectives, the K-10 was conceived as a transdiagnostic measure capturing a latent factor of general emotional vulnerability, enabling the assessment of the degree of psychological impairment experienced by an individual without being restricted to specific diagnostic categories. This approach has contributed to its widespread adoption in epidemiological and psychosocial research investigating mental health across diverse cultural contexts.

Accumulated international evidence provides strong support for the validity and reliability of the K-10 across different cultural settings, age groups, and both clinical and non-clinical populations. Studies conducted in Australia, Spain, the United States, Canada, the Netherlands, Brazil, and Iran have consistently supported a predominantly unidimensional factor structure, accompanied by high levels of internal consistency, typically exceeding α = 0.80 (Furukawa et al., 2003; Fassaert et al., 2009; Lins et al., 2021). Furthermore, the scale has demonstrated consistent associations with measures of anxiety, depression, quality of life, and subjective well-being. These characteristics have facilitated its extensive use in epidemiological and university mental health research as a brief dimensional measure of psychological distress.

Psychometric evidence on the K-10 in Spanish-speaking populations remains fragmented. The Argentine adaptation supported a dominant general factor in a community sample, whereas a two-factor solution emerged in a clinical subsample (Brenlla & Aranguren, 2010); research in Mexico reported high internal consistency and criterion performance for detecting depression and anxiety in primary care (Vargas Terrez et al., 2011); and a study of Peruvian university students supported a one-factor solution (Arias Gallegos et al., 2019).

In Ecuador, a nine-item form was supported and invariance was examined by gender rather than across countries (Larzabal-Fernandez et al., 2024), while evidence from Spain supported the unidimensional 10-item model (Sánchez-Moreno et al., 2025). Evidence from Brazilian higher education students concerns a Portuguese-language version and therefore does not establish equivalence among Spanish-speaking national samples (Perrelli et al., 2024). To our knowledge, no previous study has directly tested cross-country measurement invariance using identical Spanish K-10 wording among university students in Chile, Colombia, and Ecuador. Accordingly, the specific novelty of the present study lies in evaluating, within the same analytical framework, the scale’s unidimensional structure, item loadings, reliability, convergent validity, and configural, metric, scalar, and strict invariance across 1258 students from 14 universities in these three countries, thereby providing evidence on whether cross-national comparisons rest on a common measurement structure.

Despite its widespread use and substantial empirical support, there remains a need to further examine the psychometric equivalence of the scale across different sociocultural contexts. The absence of measurement invariance studies may limit the interpretation of cross-group comparisons, as observed differences may reflect measurement bias rather than genuine differences in the underlying construct. In this context, measurement invariance analyses are of particular importance in cross-cultural research, especially in regions such as Latin America, which are characterised by considerable social, educational, and economic heterogeneity. Establishing configural, metric, scalar, and strict invariance is essential to ensure that the K-10 measures the same construct equivalently across different countries, thereby enabling valid cross-national comparisons and strengthening the available psychometric evidence for the region (Putnick & Bornstein, 2016; Davidov et al., 2014).

Although previous validations including evidence from Brazil have established the K-10’s psychometric performance within specific national and population contexts, they do not establish whether scores can be interpreted equivalently across university students from Chile, Colombia, and Ecuador. The specific gap addressed by this study is therefore the limited directly comparable cross-national evidence concerning the scale’s internal structure, reliability, convergent validity, and successive levels of measurement invariance in these three Latin American higher-education contexts. Accordingly, the research question was: Does the K-10 demonstrate a unidimensional structure, adequate internal consistency and convergent validity, and configural, metric, scalar, and strict invariance across Chilean, Colombian, and Ecuadorian university students?

## 2. Materials and Methods

### 2.1. Participants

The analytical sample comprised 1258 university students enrolled at 14 higher education institutions in Chile, Colombia and Ecuador. Five Chilean, five Colombian and four Ecuadorian universities participated; overall, seven institutions were public and seven were private. A non-probability convenience sampling strategy was used.

The eligibility criteria were enrolment at one of the participating universities, being between 18 and 29 years of age, and providing informed consent. Participation was voluntary, and no incentives were offered. Inclusion in the analytical sample additionally required responses to all questionnaire items.

Data were collected using the QuestionPro platform (QuestionPro Inc., Austin, TX, USA; https://www.questionpro.com/), which was configured to allow participants to skip items when completing the questionnaire. Following data collection, a completeness filter was applied to select only questionnaires containing responses to all items and automatically exclude records with missing responses from the analytical dataset. Consequently, the absence of missing data in the final sample resulted from a selection procedure applied after data collection.

Of the 1340 students who accessed the questionnaire, 1258 provided complete records and constituted the analytical sample. The remaining 82 cases did not meet the completeness criterion and were excluded from the analyses. The proportion of complete questionnaires among students who accessed the survey was 93.9% (1258/1340). This indicator refers to the availability of responses to all items, given that the platform allowed participants to submit the questionnaire with unanswered items. An overall recruitment response rate was not calculated because a verifiable record of the total number of students invited was unavailable.

The Chilean sample comprised 456 students, of whom 35.5% were men and 64.5% were women, with a mean age of 20.67 years (SD = 2.29). The Colombian sample comprised 319 students, of whom 36.4% were men and 63.6% were women, with a mean age of 22.16 years (SD = 3.23). The Ecuadorian sample comprised 483 students, of whom 50.7% were men and 49.3% were women, with a mean age of 21.60 years (SD = 3.28). Across all three countries, participants’ ages ranged from 18 to 29 years.

Statistically significant differences between the national samples were observed in sex distribution, χ^2^(2, N = 1258) = 27.08, *p* < 0.001, Cramér’s V = 0.147, and mean age, F(2, 1255) = 25.73, *p* < 0.001, η^2^ = 0.039. These differences in sociodemographic composition should be considered when interpreting cross-country comparisons, as the observed differences cannot be attributed exclusively to the national context. Accordingly, the comparisons are restricted to the participating samples and do not necessarily reflect differences between the university student populations of the respective countries.

The non-probability recruitment strategy and the inclusion of complete questionnaires only may introduce selection bias. Specifically, participants who answered all items may differ from those who omitted responses in their sociodemographic characteristics or the variables under investigation. Therefore, the absence of missing data in the analytical dataset does not eliminate the possibility of bias associated with complete-case selection, and the findings should be interpreted in light of this limitation.

The sociodemographic characteristics of the study population by country are summarised in Table 1.

### 2.2. Instrument

Data were collected using a structured questionnaire comprising three sections. The first section included questions designed to characterise participants according to sociodemographic variables, including sex, age, place of residence, and socioeconomic status, among others.

The second section included the Kessler Psychological Distress Scale (K-10), the instrument under validation in the present study. The K-10 is a self-report measure designed to assess non-specific symptoms of psychological distress experienced during the previous 30 days. The scale was developed by Kessler et al. (2002) and consists of 10 items assessing manifestations primarily associated with symptoms of anxiety and depression in the general population.

Responses are recorded on a five-point Likert scale ranging from 1 (Never) to 5 (Always), with higher scores indicating greater levels of psychological distress. The total score is calculated by summing responses across the 10 items, yielding a possible score ranging from 10 to 50.

Published K-10 severity cut-offs are not based on a single international standard. The frequently used four-category scheme was derived from Australian population data and was not developed as a universally valid diagnostic classification (Andrews & Slade, 2001; Australian Bureau of Statistics, 2003). Because these thresholds originate in an English-speaking, high-income setting, their direct transfer to Chilean, Colombian, and Ecuadorian university students requires caution: sensitivity and specificity can vary according to language, age, sociocultural context, and the criterion diagnosis employed. Consistent with this concern, a validation study among Brazilian university students identified a score greater than 21 as the optimal screening threshold (sensitivity = 85.2%; specificity = 82.9%), whereas research in Ecuadorian adolescents supported a modified nine-item version and did not establish a diagnostic cut-off for university students (Perrelli et al., 2024; Larzabal-Fernandez et al., 2024). Therefore, the present study analysed K-10 scores dimensionally and did not use the Australian severity bands to classify participants, estimate disorder prevalence, or make clinical inferences. Country- and population-specific cut-offs should be established against structured diagnostic interviews through ROC analyses before clinical screening use in Chile, Colombia, or Ecuador.

The Spanish wording administered in the present study was taken from the Spanish-language K-10 reported by Sánchez-Moreno et al. (2025), which had previously been used and psychometrically evaluated in Spain. Because the source instrument was already in Spanish, the present study did not perform a new forward translation from English or a back-translation.

The task was therefore regional cross-cultural harmonisation rather than de novo translation. The same ten-item wordings, common 30-day stem, and response options were administered without modification in Chile, Colombia, and Ecuador. Supplementary Materials reproduces the exact Spanish wording presented to participants.

The third section included the Satisfaction with Life Scale (SWLS) developed by Diener et al. (1985), a five-item instrument that measures the cognitive dimension of subjective well-being through a unidimensional factor structure. Participants respond using a Likert-type scale, with higher scores indicating greater life satisfaction. Recent studies involving Latin American university students have provided evidence supporting its validity, reliability, and measurement invariance for cross-cultural comparisons (Norambuena-Paredes et al., 2025).

### 2.3. Procedure

To administer the instrument, the research team contacted the directors of the participating universities in Chile, Colombia, and Ecuador, who granted the necessary institutional authorisation for data collection at their respective sites. The full multicountry protocol was reviewed and approved by the Ethics Committee of the University of La Frontera (UFRO File No. 145/23), which served as the coordinating ethics committee and covered the common protocol implemented at all participating sites. In Colombia and Ecuador, local oversight consisted of institutional authorisation from each participating university rather than separate review by local ethics committees. Data were collected using an online questionnaire hosted on the QuestionPro platform. Participants were invited via email by the research team, with each invitation including the informed consent form and a link to the questionnaire. The informed consent document outlined the study objectives, the voluntary nature of participation, and the confidentiality and anonymity of the data. The protocol was classified as involving minimal risk; nevertheless, the consent form acknowledged that questions about psychological distress could cause mild or temporary discomfort and informed participants of their right to withdraw from the study at any time without penalty.

No pilot study was conducted prior to the main data collection. Completion of the full online questionnaire, including the initial explanation of the study, informed consent, sociodemographic questions, and all study instruments, required approximately 15 min. The questionnaire was organised into three substantive sections; however, the exact number of QuestionPro screens was not recorded. No incentives were provided to participants. The study records do not document the use of an additional technical restriction specifically designed to prevent multiple submissions; participation was voluntary and the survey link was distributed through the established university recruitment channels.

In addition, three expert judges from Chile, Colombia, and Ecuador reviewed the items of the Kessler Psychological Distress Scale (K-10) to identify and, where necessary, adapt any potentially colloquial expressions. Following this review, the experts concluded that the scale would be readily understood across the different cultural contexts included in the study. Consequently, no modifications were required, and the original version of the instrument was administered to participants in Chile, Colombia, and Ecuador.

Before administration, the Spanish K-10 underwent a targeted cross-cultural review. The panel comprised three university academics—one from Chile, one from Colombia, and one from Ecuador—with research experience in psychometrics, instrument validation, and higher-education contexts. Each judge assessed the items according to four predefined criteria: semantic clarity, comprehensibility for university students, linguistic and cultural appropriateness, and preservation of the intended meaning of psychological distress.

The judges first reviewed the common Spanish form from their country-specific perspective and then reached consensus. They agreed that all ten items and response options were clear and culturally appropriate in the three settings; consequently, no wording changes were made and exactly the same form was administered in each country. This procedure was a qualitative cultural-suitability review of an existing Spanish version, not a new translation study. Because decisions were consensus-based, no quantitative content-validity index or inter-rater agreement coefficient was calculated. Data collection was carried out simultaneously across the three countries during June 2025.

### 2.4. Data Analysis

Statistical analyses were conducted using SPSS version 25 and Mplus version 8.11. Initially, descriptive statistics were calculated for each Kessler Psychological Distress Scale (K-10) item, including means, standard deviations, skewness, and kurtosis coefficients.

The factorial structure of the K-10 was examined using confirmatory factor analysis (CFA). Given the ordered categorical nature of the items, models were estimated using the Weighted Least Squares Mean and Variance Adjusted (WLSMV) estimator with THETA parameterization. Model fit was evaluated using the Comparative Fit Index (CFI) and Tucker–Lewis Index (TLI), with values ≥ 0.90 indicating acceptable fit and ≥0.95 a good fit, and the Root Mean Square Error of Approximation (RMSEA) and Standardized Root Mean Square Residual (SRMR), with values ≤ 0.08 considered acceptable and ≤0.05 indicative of close fit (Browne & Cudeck, 1993; Hu & Bentler, 1999).

The initial unidimensional CFA retained the original five ordered response categories. Because this model showed inadequate fit according to several indices, sensitivity analyses were conducted using alternative category groupings while retaining the same factorial structure. Two three-category specifications (1–2/3/4–5 and 1–3/4/5) and two binary specifications (1–2/3–5 and 1–3/4–5) were examined. As these specifications involved different transformations of the observed responses, their fit was compared descriptively rather than through nested-model difference testing. The 1–2/3–5 binary specification was retained for subsequent analyses based on its model fit and substantive interpretability. The retained model was subsequently evaluated separately in each country.

Measurement invariance across Chile, Colombia, and Ecuador was examined using multigroup CFA with WLSMV estimation and THETA parameterization. Given the binary specification retained for the K-10 items, measurement invariance was evaluated through configural and scalar models, following the identification framework for categorical indicators implemented in Mplus (Muthén & Muthén, 2017). Each item was therefore represented by a single threshold. Configural invariance was first evaluated by specifying the same unidimensional structure across groups. Scalar invariance was subsequently tested by constraining factor loadings and item thresholds to equality across countries. Invariance was primarily evaluated using changes in approximate fit indices, with ΔCFI ≤ 0.010 and ΔRMSEA ≤ 0.015 indicating invariance (Chen, 2007). The WLSMV DIFFTEST was additionally examined for nested-model comparison. Complete Mplus syntax and relevant output are provided in the Supplementary Material.

Following support for scalar invariance, latent means were compared across countries, with Ecuador specified as the reference group. Pairwise contrasts were estimated with 95% confidence intervals, and *p*-values were adjusted using the Holm procedure. Because latent factor variances were fixed to one, latent mean differences were expressed in standard deviation units.

Evidence of convergent validity was examined separately in each country using latent correlations between psychological distress and life satisfaction. Two-factor models were estimated in Mplus, with the binary K-10 items loading on the psychological distress factor and the ordinal Satisfaction with Life Scale (SWLS) items loading on the life satisfaction factor. Models were estimated using WLSMV with THETA parameterization, and standardized latent correlations with 95% confidence intervals were reported.

Finally, internal consistency was evaluated separately in each country using ordinal alpha (α), based on tetrachoric correlations, and McDonald’s omega (ω), derived from the categorical CFA model. Coefficients ≥ 0.70 were considered indicative of satisfactory internal consistency (Trizano-Hermosilla et al., 2021).

## 3. Results

### 3.1. Descriptive Analyses

Table 2 summarises the descriptive statistics for the ten K-10 items across the Chilean, Colombian, and Ecuadorian samples. Mean scores were relatively comparable across the three countries, suggesting similar item-level response patterns. Regarding the skewness and kurtosis coefficients, there was relatively limited variation among the three samples, with skewness ranging from −0.13 to 0.37 and kurtosis from −0.68 to 0.35, indicating no pronounced skewness or extreme concentration of responses. Overall, the descriptive results revealed comparable distribution patterns among the three national samples.

### 3.2. Factor Structure of the Kessler Psychological Distress Scale (K-10)

The quality of the unidimensional model was evaluated using multiple goodness-of-fit indices. With the original five response categories, the model showed mixed evidence of fit: SRMR was adequate (0.046), whereas CFI (0.921), TLI (0.898), and particularly RMSEA (0.154) indicated insufficient fit. The category thresholds were correctly ordered across items, suggesting that the observed misfit was not attributable to disordered thresholds.

To examine the robustness of the factor structure to the treatment of the response categories, a sensitivity analysis was conducted using alternative category groupings while retaining the same unidimensional model. The alternative category specifications showed different levels of model fit, with the binary specifications providing the most consistent results. Both the 1–2/3–5 solution (CFI = 0.972, TLI = 0.964, RMSEA = 0.065, SRMR = 0.057) and the alternative 1–3/4–5 solution (CFI = 0.968, TLI = 0.959, RMSEA = 0.068, SRMR = 0.059) provided adequate fit.

The 1–2/3–5 specification was retained because it combined adequate fit with a substantively interpretable distinction between lower-frequency responses (never/almost never) and at-least-occasional symptom responses (sometimes/almost always/always). Separate CFAs subsequently supported the unidimensional structure in Chile (CFI = 0.972, TLI = 0.964, RMSEA = 0.057, SRMR = 0.067), Colombia (CFI = 0.972, TLI = 0.964, RMSEA = 0.069, SRMR = 0.077), and Ecuador (CFI = 0.978, TLI = 0.972, RMSEA = 0.063, SRMR = 0.060). Standardized factor loadings were positive and statistically meaningful across countries, ranging from 0.439 to 0.832 in Chile, 0.642 to 0.860 in Colombia, and 0.619 to 0.863 in Ecuador. All 95% confidence intervals excluded zero (Table 3).

### 3.3. Measurement Invariance

Measurement invariance of the retained K-10 model was examined across Chile, Colombia, and Ecuador. The configural model showed adequate fit (χ^2^(105) = 275.79, CFI = 0.976, TLI = 0.969, RMSEA = 0.062, SRMR = 0.067), supporting a common unidimensional structure across countries. The scalar model, in which factor loadings and item thresholds were constrained to equality across groups, also showed adequate fit (χ^2^(121) = 291.76, CFI = 0.976, TLI = 0.973, RMSEA = 0.058, SRMR = 0.069). (See Table 4).

Changes in approximate fit indices were minimal (ΔCFI = 0.000, ΔRMSEA = −0.004, ΔSRMR = 0.002), providing support for scalar measurement invariance. Although the WLSMV DIFFTEST was marginally significant, Δχ^2^(16) = 26.41, *p* = 0.049, changes in the approximate fit indices remained within the prespecified criteria. Taken together, these results supported scalar invariance across the three national samples.

### 3.4. Evidence of Convergent Validity

Evidence of convergent validity was examined by assessing the theoretically expected inverse association between psychological distress, measured using the Kessler Psychological Distress Scale (K-10), and life satisfaction, measured using the Satisfaction with Life Scale (SWLS). In the Chilean sample, a moderate negative latent correlation was found between psychological distress and life satisfaction (r = −0.485, *p* < 0.001, 95% CI [−0.570, −0.400]). Similarly, the Colombian sample showed a significant inverse association, although of smaller magnitude (r = −0.274, *p* < 0.001, 95% CI [−0.399, −0.149]). In the Ecuadorian sample, the association was also negative and statistically significant, but small in magnitude (r = −0.119, *p* = 0.048, 95% CI [−0.236, −0.001]). Overall, these findings provide evidence of the expected inverse association between psychological distress and life satisfaction across the three countries.

### 3.5. Evidence of Reliability

Following confirmation of the unidimensional factor structure and measurement invariance across countries, the internal consistency of the Kessler Psychological Distress Scale (K-10) was evaluated separately in each national sample. Given the binary response specification retained for the items, internal consistency was estimated using coefficients based on tetrachoric correlations. The results indicated high internal consistency across the three samples. Ordinal McDonald’s omega was 0.910 in Chile, 0.929 in Colombia, and 0.936 in Ecuador. Corresponding ordinal alpha coefficients were 0.903, 0.923, and 0.932, respectively.

## 4. Discussion

The present study provides evidence regarding the psychometric properties of the K-10 among university students from Chile, Colombia, and Ecuador. The results supported a unidimensional structure for the binary solution, together with scalar invariance and high internal consistency. This level of equivalence enabled comparisons of latent means across the three national samples. However, the conclusions are restricted to the recoding examined and cannot be directly extended to the original five-category format.

The original model showed mixed evidence of fit, despite the thresholds remaining correctly ordered, indicating that its inadequate performance was not explained by disordered categories. The binary solution demonstrated better fit indices and factor loadings ranging from 0.439 to 0.863, with confidence intervals excluding zero. These findings are consistent with the conceptualisation of the K-10 as a measure of a general factor of psychological distress (Kessler et al., 2002). They are also consistent with Latin American research identifying a relevant general factor within its internal structure (Arias Gallegos et al., 2019; Perrelli et al., 2024).

The dichotomous use of the K-10 items has some empirical precedent, although it remains less common than the use of its ordinal scores. Mousley et al. (2015) dichotomised the ten items into symptom absence and symptom presence experienced ‘a little or more’ to compare adult population groups in Ethiopia. The present study applied a more conservative criterion by distinguishing between never/almost never and a frequency of at least sometimes. This precedent supports the feasibility of analysing the items dichotomously, but does not demonstrate that the two recoding schemes are equivalent or that the cut-off used in the present study is optimal.

Configural invariance indicated that the unidimensional organisation of psychological distress was similar across the three countries, whereas scalar invariance supported the equality of factor loadings and thresholds. Changes in the CFI, RMSEA, and SRMR remained within the criteria recommended by Chen (2007), with no evidence of a substantive deterioration in model fit. Although the DIFFTEST was marginally significant, this finding should be interpreted alongside the approximate fit indices, given the sensitivity of the chi-square statistic to sample size (Davidov et al., 2014; Putnick & Bornstein, 2016). The evidence obtained permits comparisons of latent means, but does not establish strict invariance or equivalence of the original response format.

Latent comparisons showed higher psychological distress in the Ecuadorian sample than in the Chilean and Colombian samples. The differences were 0.356 and 0.377 standard deviations, respectively, and remained statistically significant after the Holm correction; by contrast, Chile and Colombia did not differ significantly. These magnitudes may be regarded as small to moderate and should be distinguished from their statistical significance (Cohen, 1988). Academic, economic, social, and environmental factors might contribute to these patterns (Aghabozorgi et al., 2024; Cassaretto et al., 2021), but they were not examined and therefore cannot support country-level explanations.

The inverse correlations between psychological distress and life satisfaction provided convergent validity evidence in the theoretically expected direction. The association was moderate in Chile, small in Colombia, and weak in Ecuador, suggesting that the relationship between the two constructs did not display the same descriptive magnitude across all samples (Diener et al., 1985; Peixoto & Cunha, 2024). Similarly, the omega and ordinal alpha coefficients indicated high internal consistency across the three countries (Trizano-Hermosilla et al., 2021). Taken together, these results support the dimensional use of the binary solution in research, but do not justify its clinical application or the use of diagnostic cut-offs.

The results should be interpreted in light of several limitations. The non-probability sampling method and the participation of specific institutions restrict generalisation to the national university populations, while the cross-sectional design precludes conclusions about causal relationships or changes over time. The exclusive inclusion of complete questionnaires and the use of self-report measures may have introduced selection and common method biases. In addition, the binary solution was selected using the same dataset in which it was evaluated, lacked independent cross-validation, reduced information about symptom frequency, and included life satisfaction as the sole external criterion.

Future research should replicate the model in probabilistic, independent samples balanced across countries, sexes, and types of institution. It would also be valuable to compare the different category groupings using item response models, information curves, and differential item functioning analyses to determine which recoding retains the greatest measurement precision. Longitudinal studies should examine temporal stability, sensitivity to change, and predictive validity while incorporating clinical, academic, and social indicators. Before this version is used for screening, locally appropriate cut-offs must be established and its sensitivity, specificity, and diagnostic accuracy evaluated (Andrews & Slade, 2001; Kessler et al., 2003).

## 5. Conclusions

The binary solution of the K-10 demonstrated a unidimensional structure, scalar invariance, and high internal consistency among university students from Chile, Colombia, and Ecuador. In addition, the inverse associations with life satisfaction provided evidence of convergent validity in all three countries. The level of equivalence achieved enabled comparisons of latent means, revealing higher psychological distress in the Ecuadorian sample than in the Chilean and Colombian samples. These findings support the use of the version examined in cross-cultural research and for the group-level description of psychological wellbeing among university students.

In the context of student welfare policies, the K-10 may help identify differences across university communities, guide resource allocation, and inform preventive mental health programmes. Its periodic administration at an aggregate level could also support the assessment of students’ needs and the design of psychological, academic, and social support strategies tailored to each context. However, the findings do not yet support its use for individual screening, clinical decision-making, or intervention evaluation. Such applications require further research on locally appropriate cut-off scores, sensitivity to change, and diagnostic accuracy.

## Figures and Tables

**Table 1:** Sociodemographic characteristics of the study population by country.

Characteristic	Chile	Colombia	Ecuador	Total
Sample size, n	456	319	483	1258
Age, M (SD)	20.67 (2.29)	22.16 (3.23)	21.60 (3.28)	21.40 (3.00)
Age range, years	18–29	18–29	18–29	18–29
Men, n (%)	162 (35.5)	116 (36.4)	245 (50.7)	523 (41.6)
Women, n (%)	294 (64.5)	203 (63.6)	238 (49.3)	735 (58.4)
Belongs to an ethnic group, n (%)	106 (23.2)	12 (3.8)	57 (11.8)	175 (13.9)
Does not belong to an ethnic group, n (%)	350 (76.8)	307 (96.2)	426 (88.2)	1083 (86.1)
Universities, n	5	5	4	14

**Note.** Values are presented as M (SD) for age and n (%) for sex. The overall age mean and standard deviation were derived from the country-specific summary statistics. Participants were recruited from 14 universities (five in Chile, five in Colombia, and four in Ecuador); seven institutions were public and seven were private.

**Table 2:** Descriptive statistics of K-10 items across countries.

Items	M	SD	g_1_	g_2_
**Chilean Sample (n = 456)**				
Item 1	3.63	0.84	−0.13	−0.14
Item 2	3.48	0.87	0.07	−0.59
Item 3	2.65	1.02	0.18	−0.28
Item 4	2.67	1.01	0.25	−0.13
Item 5	3.22	0.85	0.12	0.16
Item 6	2.79	0.98	0.23	−0.11
Item 7	3.01	0.92	0.07	0.08
Item 8	3.36	0.90	0.13	−0.27
Item 9	2.61	0.94	0.21	−0.09
Item 10	2.86	1.12	0.07	−0.55
**Colombian Sample (n = 319)**				
Item 1	3.43	0.88	−0.13	0.24
Item 2	3.22	0.90	0.05	0.02
Item 3	2.58	1.04	0.37	−0.29
Item 4	2.67	1.03	0.16	−0.30
Item 5	2.97	0.88	0.09	0.29
Item 6	2.71	0.99	0.04	−0.30
Item 7	2.83	0.98	0.01	−0.03
Item 8	3.25	0.98	−0.10	−0.31
Item 9	2.64	1.00	0.23	−0.26
Item 10	2.69	1.15	0.16	−0.68
**Ecuadorian Sample (n = 483)**				
Item 1	3.21	0.92	−0.13	0.22
Item 2	3.35	0.92	−0.02	−0.06
Item 3	2.78	1.04	0.25	−0.23
Item 4	2.83	0.97	0.08	0.03
Item 5	3.00	0.89	0.14	0.35
Item 6	2.80	0.97	0.16	0.14
Item 7	3.00	0.99	−0.03	0.02
Item 8	3.26	0.98	−0.06	−0.13
Item 9	2.80	1.00	0.21	−0.03
Item 10	2.71	1.10	0.14	−0.46

**Note.** M = mean; SD = standard deviation; g_1_ = skewness coefficient; g_2_ = kurtosis coefficient.

**Table 3:** Factor structure of the Kessler Psychological Distress Scale (K-10) across countries.

Items	Standardized Factor Loading (λ)	S.E.	95% CI for λ
**Chile**			
Item 1	0.619	0.085	[0.453, 0.785]
Item 2	0.663	0.061	[0.543, 0.782]
Item 3	0.790	0.036	[0.719, 0.860]
Item 4	0.832	0.035	[0.764, 0.900]
Item 5	0.766	0.044	[0.680, 0.852]
Item 6	0.641	0.046	[0.550, 0.732]
Item 7	0.755	0.042	[0.672, 0.837]
Item 8	0.439	0.067	[0.307, 0.570]
Item 9	0.831	0.032	[0.768, 0.894]
Item 10	0.714	0.042	[0.632, 0.797]
**Colombia**			
Item 1	0.647	0.071	[0.508, 0.786]
Item 2	0.648	0.060	[0.531, 0.766]
Item 3	0.777	0.044	[0.691, 0.863]
Item 4	0.845	0.036	[0.774, 0.916]
Item 5	0.822	0.040	[0.743, 0.901]
Item 6	0.776	0.042	[0.694, 0.859]
Item 7	0.788	0.042	[0.705, 0.871]
Item 8	0.642	0.061	[0.523, 0.761]
Item 9	0.860	0.034	[0.793, 0.927]
Item 10	0.694	0.050	[0.596, 0.792]
**Ecuador**			
Item 1	0.619	0.059	[0.504, 0.734]
Item 2	0.690	0.053	[0.586, 0.795]
Item 3	0.778	0.034	[0.714, 0.848]
Item 4	0.855	0.028	[0.799, 0.910]
Item 5	0.816	0.035	[0.747, 0.885]
Item 6	0.773	0.035	[0.706, 0.845]
Item 7	0.851	0.031	[0.789, 0.912]
Item 8	0.624	0.055	[0.516, 0.731]
Item 9	0.863	0.028	[0.809, 0.917]
Item 10	0.816	0.030	[0.757, 0.875]

**Note.** Standardized factor loadings (STDYX) are reported. S.E. = standard error; CI = confidence interval.

**Table 4:** Measurement invariance of the K-10 across Chilean, Colombian, and Ecuadorian samples.

Model	χ^2^ (df)	CFI	TLI	RMSEA	SRMR	ΔCFI	ΔRMSEA	ΔSRMR	Conclusion
Configural	275.79 (105)	0.976	0.969	0.062	0.067	—	—	—	Supported
Scalar	291.76 (121)	0.976	0.973	0.058	0.069	0.000	−0.004	0.002	Supported

Given the evidence of scalar invariance, latent means were subsequently compared across countries, with Ecuador specified as the reference group (M = 0). Because latent factor variances were fixed to 1 across groups, latent mean differences are expressed in standard deviation units. Chile showed lower latent psychological distress than Ecuador (ΔM = −0.356, SE = 0.121, 95% CI [−0.594, −0.118], *p* = 0.003), corresponding to a standardized difference of −0.356 SD. Colombia also showed lower latent distress than Ecuador (ΔM = −0.377, SE = 0.137, 95% CI [−0.646, −0.109], *p* = 0.006), corresponding to −0.377 SD. In contrast, Chile and Colombia did not differ (ΔM = 0.021, SE = 0.094, 95% CI [−0.163, 0.205], *p* = 0.821). After Holm correction, the Chile–Ecuador (adjusted *p* = 0.009) and Colombia–Ecuador (adjusted *p* = 0.012) contrasts remained statistically significant.

## Data Availability

The dataset for the study is available from the corresponding author upon reasonable request due to ethical restrictions.

## Supplementary Materials

The following supporting information can be downloaded at: https://www.mdpi.com/article/10.3390/bs16101812/s1, the original Spanish items and English version of the Kessler Psychological Distress Scale (K-10), together with the complete Mplus syntax used for the confirmatory factor analyses and measurement invariance models.
