# Supplementary material for: Measurement Invariance of the Kessler Psychological Distress Scale (K-10) Among Chilean, Colombian and Ecuadorian University Students
Source: Behav Sci (Basel). 2026 Oct 3;16(10):1812. doi: 10.3390/bs16101812 (PMC13701272; doi:10.3390/bs16101812)
Supplement: Supplementary file 1 [file behavsci-16-01812-s001.zip › behavsci-4465979-supplementary.pdf]

Material suplementario/ Supplementary Material

Ítems de la Escala de Malestar Psicológico de Kessler (K-10) utilizada en el presente estudio / Items of the Kessler Psychological Distress Scale (K-10) Used in the Present Study

Instrucciones/ **Instructions:**

A continuación, encontrará una serie de preguntas relacionadas con cómo se ha sentido durante los últimos 30 días. Por favor, lea cada afirmación cuidadosamente e indique con qué frecuencia ha experimentado cada situación, marcando solo una alternativa para cada pregunta / **Below are a number of questions about how you have been feeling during the past 30 days. Please read each question carefully and indicate how often you experienced each situation by selecting one response for each item.**

Versión original en español / **Original version in Spanish**

		Nunca	Casi nunca	A veces	Casi siempre	Siempre
1	¿Con qué frecuencia se ha sentido cansado/a, sin tener una buena razón	☐	☐	☐	☐	☐
2	¿Con qué frecuencia se ha sentido nervioso/a?	☐	☐	☐	☐	☐
3	¿Con qué frecuencia se ha sentido tan nervioso/a que nada podía calmarle?	☐	☐	☐	☐	☐
4	¿Con qué frecuencia se ha sentido sin esperanza?	☐	☐	☐	☐	☐
5	¿Con qué frecuencia se ha sentido inquieto/a o intranquilo/a?	☐	☐	☐	☐	☐
6	¿Con qué frecuencia se ha sentido tan impaciente que no podía mantenerse quieto/a o sentado/a?	☐	☐	☐	☐	☐

7	¿Con qué frecuencia se ha sentido deprimido/a?	☐	☐	☐	☐	☐
8	¿Con qué frecuencia ha sentido que todo lo que hace supone un gran esfuerzo?	☐	☐	☐	☐	☐
9	¿Con qué frecuencia se ha sentido tan triste que nada podía animarle?	☐	☐	☐	☐	☐
10	¿Con qué frecuencia se ha sentido inútil?	☐	☐	☐	☐	☐

Versión en inglés / English version

		Nunca	Casi nunca	A veces	Casi siempre	Siempre
1	How often did you feel tired out for no good reason?	☐	☐	☐	☐	☐
2	How often did you feel nervous?	☐	☐	☐	☐	☐
3	How often did you feel so nervous that nothing could calm you down?	☐	☐	☐	☐	☐
4	How often did you feel hopeless?	☐	☐	☐	☐	☐
5	How often did you feel restless or fidgety?	☐	☐	☐	☐	☐

6	How often did you feel so restless that you could not sit still?	☐	☐	☐	☐	☐
7	How often did you feel depressed?	☐	☐	☐	☐	☐
8	How often did you feel that everything was an effort?	☐	☐	☐	☐	☐
9	How often did you feel so sad that nothing could cheer you up?	☐	☐	☐	☐	☐
10	How often did you feel worthless?	☐	☐	☐	☐	☐

Supplementary Material S1. Mplus syntax used for confirmatory factor analysis and cross-national measurement invariance testing of the K-10

All categorical models were estimated in Mplus version 8.11 using the WLSMV estimator with THETA parameterization. Missing values were coded as 99.

A. Original five-category CFA

```
TITLE: K-10 CFA - original five ordered categories;
DATA: FILE = B1.dat;
VARIABLE:
  NAMES = ID Edad Sexo Nacionalidad Etnia
        it1SWLS-it5SWLS it1ApoyoSocial-it6ApoyoSocial
        it1K10-it10K10;
  USEVARIABLES = it1K10-it10K10;
  CATEGORICAL = it1K10-it10K10;
  MISSING = ALL (99);
ANALYSIS:
  ESTIMATOR = WLSMV;
  PARAMETERIZATION = THETA;
MODEL:
  K10 BY it1K10-it10K10;
OUTPUT: STDYX CINTERVAL TECH1;
```

B. Retained binary CFA (original categories 1-2 vs. 3-5)

```
TITLE: K-10 CFA - retained binary specification;
DATA: FILE = B2.dat;
VARIABLE:
```

```
  NAMES = ID Edad Sexo Nacionalidad Etnia
        it1SWLS-it5SWLS it1ApoyoSocial-it6ApoyoSocial
        it1K10-it10K10;
  USEVARIABLES = it1K10-it10K10;
  CATEGORICAL = it1K10-it10K10;
  MISSING = ALL (99);
ANALYSIS:
  ESTIMATOR = WLSMV;
  PARAMETERIZATION = THETA;
MODEL:
  K10 BY it1K10-it10K10;
OUTPUT: STDYX CINTERVAL TECH1;
```

C. Country-specific CFA

The retained binary CFA was estimated separately by changing the USEOBSERVATIONS statement to Nacionalidad EQ 1 (Chile), EQ 2 (Colombia), and EQ 3 (Ecuador).

```
VARIABLE:
  NAMES = ID Edad Sexo Nacionalidad Etnia
        it1SWLS-it5SWLS it1ApoyoSocial-it6ApoyoSocial
        it1K10-it10K10;
  USEVARIABLES = it1K10-it10K10;
  USEOBSERVATIONS = Nacionalidad EQ 1;
  CATEGORICAL = it1K10-it10K10;
  MISSING = ALL (99);
ANALYSIS:
  ESTIMATOR = WLSMV;
  PARAMETERIZATION = THETA;
MODEL:
  K10 BY it1K10-it10K10;
OUTPUT: STDYX CINTERVAL TECH1;
```

D-E. Configural and scalar measurement invariance

Because the retained indicators were binary, each item contributed one threshold. The configural model specified the same one-factor structure across groups. In the scalar model, factor loadings and the single threshold of each item were constrained to equality across countries. A separate strict-invariance model was not retained for the binary THETA specification.

```
TITLE: K-10 measurement invariance - Chile, Colombia, Ecuador;
DATA: FILE = B2.dat;
VARIABLE:
  NAMES = ID Edad Sexo Nacionalidad Etnia
        it1SWLS-it5SWLS it1ApoyoSocial-it6ApoyoSocial
        it1K10-it10K10;
  USEVARIABLES = it1K10-it10K10;
  CATEGORICAL = it1K10-it10K10;
  MISSING = ALL (99);
  GROUPING = Nacionalidad (1 = CHI 2 = COL 3 = ECU);
ANALYSIS:
  ESTIMATOR = WLSMV;
  PARAMETERIZATION = THETA;
  MODEL = CONFIGURAL SCALAR;
```

```
MODEL:
  K10 BY it1K10-it10K10;
OUTPUT: STDYX CINTERVAL TECH1;
```

Selected Mplus output for verification

```
Number of observations
  Chile       456
  Colombia    319
  Ecuador     483
  Total      1258

CONFIGURAL MODEL
  Number of free parameters = 60
  Chi-Square = 275.794, df = 105
  CFI = .976   TLI = .969
  RMSEA = .062, 90% CI [.053, .071]
  SRMR = .067

SCALAR MODEL
  Number of free parameters = 44
  Chi-Square = 291.764, df = 121
  CFI = .976   TLI = .973
  RMSEA = .058, 90% CI [.050, .067]
  SRMR = .069

WLSMV DIFFTEST: scalar versus configural
  Delta Chi-Square(16) = 26.414, p = .0485

Changes in approximate fit
  Delta CFI = .000
  Delta RMSEA = -.004
  Delta SRMR = .002
```

In the scalar model, the ten factor loadings and the ten binary-item thresholds were constrained to equality across the three groups. The reduction from 60 to 44 free parameters corresponds to the additional cross-group equality restrictions under the categorical identification used by Mplus.

F. Latent mean comparisons

```
TITLE: K-10 scalar invariance with latent mean comparisons;
DATA: FILE = B2.dat;
VARIABLE:
  NAMES = ID Edad Sexo Nacionalidad Etnia
        it1SWLS-it5SWLS it1ApoyoSocial-it6ApoyoSocial
        it1K10-it10K10;
  USEVARIABLES = it1K10-it10K10;
  CATEGORICAL = it1K10-it10K10;
  MISSING = ALL (99);
  GROUPING = Nacionalidad (1 = CHI 2 = COL 3 = ECU);
ANALYSIS:
  ESTIMATOR = WLSMV;
  PARAMETERIZATION = THETA;
MODEL:
```

```
  K10 BY it1K10-it10K10;
MODEL CHI:
  [K10];
MODEL COL:
  [K10];
MODEL ECU:
  [K10@0];
OUTPUT: STDYX CINTERVAL TECH1;
```

Ecuador was the reference group (latent mean fixed to 0). Pairwise contrasts were evaluated from the fitted scalar model and p-values were adjusted using the Holm procedure.

G. K-10-SWLS latent correlations

```
TITLE: K-10-SWLS convergent validity - country-specific model;
DATA: FILE = <country data file>;
VARIABLE:
  NAMES = ID Edad Sexo Nacional Etnia
        SWLS1-SWLS5 AS1-AS6 K1-K10;
  USEVARIABLES = SWLS1-SWLS5 K1-K10;
  CATEGORICAL = SWLS1-SWLS5 K1-K10;
  MISSING = ALL (99);
ANALYSIS:
  ESTIMATOR = WLSMV;
  PARAMETERIZATION = THETA;
MODEL:
  SWLS BY SWLS1-SWLS5;
  DISTRESS BY K1-K10;
  SWLS WITH DISTRESS;
OUTPUT: STDYX CINTERVAL TECH1;
```

This model was estimated separately in Chile, Colombia, and Ecuador. Standardized latent correlations (STDYX) and 95% confidence intervals were reported.

Reliability note

For the retained binary specification, ordinal alpha was calculated from tetrachoric correlations, and model-based McDonald's omega was derived from the standardized loadings of the categorical CFA model.
